# Disrupted Vaginal Microbiota and Increased HPV Infection Risk Among Non-Vaccinated Women: Findings from a Prospective Cohort Study in Kazakhstan

**DOI:** 10.3390/vaccines13070679

**Published:** 2025-06-25

**Authors:** Kuralay Kongrtay, Kuat Kassymbek, Gulzhanat Aimagambetova, Nazira Kamzayeva, Sanimkul Makhambetova, Makhabbat Galym, Zhanar Abdiyeva, Milan Terzic, Kadisha Nurgaliyeva, Talshyn Ukybassova

**Affiliations:** 1 Department of Surgery, School of Medicine, Nazarbayev University, Astana 010000, Kazakhstan; kkongrtay@nu.edu.kz (K.K.); gulzhanat.aimagambetova@nu.edu.kz (G.A.); milan.terzic@nu.edu.kz (M.T.); 2 Clinical Academic Department of Women’s Health, CF “University Medical Center”, Astana 010000, Kazakhstan; kuat.kassymbek@alumni.nu.edu.kz (K.K.); sanai.nadirova@gmail.com (S.M.); mahabbat_g@mail.ru (M.G.); talshynu@yandex.ru (T.U.); 3 Clinical Academic Department of Laboratory Medicine, Pathology and Genetics, CF “University Medical Center”, Astana 010000, Kazakhstan; abdievaz@list.ru (Z.A.); kadinur@mail.ru (K.N.)

**Keywords:** HPV, STI, CIN, CC, vaginal microbiota, bacterial vaginosis, genital tract microecological disorders

## Abstract

**Introduction**: Vaginal microbiota has emerged as an important factor influencing human papillomavirus (HPV) persistence and host immunity. While HPV infection is often transient, persistent infections with high-risk HPV genotypes significantly increase the risk of cervical carcinogenesis. Thus, this study aims to investigate the association between microflora/sexually transmitted infections (STIs) and HPV infection, with a focus on the prevalence of coinfection and the potential role of genital tract microecological disorders. **Methods**: A prospective cohort study was conducted at a tertiary care center in Astana, Kazakhstan, between November 2024 and March 2025. A total of 396 non-pregnant women aged 18–45 years were enrolled during routine gynecological screening. Cervical samples were collected for high-risk HPV genotyping and the detection of 11 other vaginal microorganisms using real-time PCR. **Results:** HPV-positive women were significantly younger and more likely to be single compared to HPV-negative participants. They also had fewer pregnancies and deliveries and were more likely to use barrier contraception. Among STIs, *Mycoplasma hominis* demonstrated a significant association with HPV infection (adjusted OR = 2.16, 95% CI: 1.15–4.05, *p* = 0.017). Overall STI presence (adjusted OR = 2.16, *p* = 0.017) and STI multiplicity (adjusted OR = 1.36 per additional STI, *p* = 0.017) were also significantly associated with HPV positivity. Correlation analysis revealed a moderate association between *Chlamydia trachomatis* and *Trichomonas vaginalis* (ϕ = 0.39, *p* < 0.001), suggesting shared ecological or transmission pathways. **Conclusion:** The findings highlight the relevance of specific vaginal pathogens, particularly *Mycoplasma hominis,* and co-infection patterns in increasing the risk of HPV infection. These results underscore the importance of comprehensive STI screening and microbial profiling in cervical cancer prevention strategies, especially in populations with limited access to HPV vaccination. Further longitudinal and mechanistic studies are warranted to elucidate causal pathways and progression to cervical neoplasia.

## 1. Introduction

The human vagina is not sterile, and an actively self-sustaining ecosystem with a complex microenvironment plays a significant role in keeping the female genital tract healthy [1]. In reproductive-age women, the healthy vaginal microbiome is predominantly colonized by *Lactobacillus* species, such as *L. crispatus*, *L. jensenii*, and *L. gasseri*, which create an acidic and protective environment by contributing to epithelial barrier functioning, promoting immune homeostasis, and supporting spontaneous clearance of transient infections [2,3,4].

Various behavioral, hormonal, and physiological factors can disrupt this balance. This dysbiosis, marked by the overgrowth of anaerobic or aerobic organisms and the depletion of Lactobacillus, can lead to genital tract microecological disorders, increasing susceptibility to both endogenous and sexually transmitted infections [5,6]. Among these co-factors, sexually transmitted infections (STIs) have emerged as key contributors to the modulation of the cervical immune environment and viral persistence. Several epidemiological and molecular studies suggest that coinfection with common STIs, including *Chlamydia trachomatis*, *Neisseria gonorrhoeae*, *Mycoplasma genitalium*, *Ureaplasma urealyticum*, *Ureaplasma parvum*, and herpes simplex virus type 2 (HSV-2) can impair local immunity, increase inflammation, and facilitate the establishment or persistence of oncogenic HPV infections [7,8]. For example, *C. trachomatis* has been associated with increased susceptibility to HPV and may disrupt epithelial integrity, while the *Mycoplasma* and *Ureaplasma* species have shown subtype-specific associations with HPV persistence and immune evasion mechanisms [9,10].

In addition to specific pathogens, bacterial vaginosis (BV) and aerobic vaginitis (AV) have been increasingly recognized as important risk factors for HPV acquisition and persistence. These conditions are characterized by the depletion of protective *Lactobacillus* spp. and the overgrowth of potentially pathogenic anaerobes or aerobes, such as *Gardnerella vaginalis*, *Prevotella* spp., and *Streptococcus agalactiae* [11,12]. This imbalance leads to chronic inflammation, elevated levels of cytokines such as IL-1β and IL-6, and increased susceptibility to viral infections [5,13]. Moderate-to-severe AV, in particular, has been linked with a significantly higher prevalence of HPV infection, potentially acting as an independent cofactor in the pathogenesis of cervical neoplasia [14,15].

In Kazakhstan, cervical cancer continues to pose a major public health burden. As of 2022, the age-standardized incidence was 19 per 100,000 women, and cervical cancer ranked as the second cancer affecting women of reproductive age [16,17,18]. Following the World Health Organization (WHO) “90-70-90” Cervical Cancer Elimination Strategy, in 2024, Kazakhstan incorporated the HPV vaccination into the national vaccination calendar. The vaccination program follows a two-dose schedule, and the target group is 11-year-old girls. As a secondary prevention for cervical cancer, the national cervical cancer screening program employs the Papanicolaou test, which is implemented for women of 30–70 years old every 4 years. However, the screening coverage remains as low as 46%, not reaching the WHO target of 70% [17]. Nevertheless, despite the implementation of primary and secondary cervical cancer prevention strategies, limited STI surveillance, low public awareness, and inconsistent screening coverage challenge the effectiveness of existing prevention approaches [19]. Considering that HPV is a STI and could lead to potential synergistic effects between HPV and other STIs, understanding the pattern of co-infection is essential for optimizing cervical cancer prevention and the management of cervical precancerous lesions [20]. However, data from Central Asia on HPV-STI coinfections’ prevalence and clinical significance remain scarce and are published in Russian. Thus, this study aims to investigate the association between vaginal microflora/STIs and HPV infection, with a focus on the prevalence of coinfection and the potential role of genital tract microecological disorders.

## 2. Material and Methods

### 2.1. Study Design and Setting

A prospective cross-sectional cohort study was conducted in the Mother and Child Center, Corporate Fund University Medical Center (UMC), a tertiary care hospital in Astana, Kazakhstan. Samples were collected from November 2024 to March 2025. The study followed the Strengthening the Reporting of Observational Studies in Epidemiology (STROBE) guidelines [21].

### 2.2. Study Subjects

All women aged between 18 and 45 years old who met the eligibility criteria and signed the informed consent form were recruited to the study. The inclusion criteria comprise women aged 18–45 years old, who were not pregnant at the time of the study, and with a regular menstrual cycle. Exclusion criteria were the following: the presence of complex concomitant chronic diseases (hepatitis B and C, diabetes mellitus, autoimmune diseases, HIV-infected and oncological diseases at present and in history) in any location; acute inflammatory processes of any localization at the time of the study; use of probiotics and/or antibiotic therapy and/or immunosuppressive therapy within the previous 14 days; smoking; intrauterine device in situ; history of HPV vaccination; any invasive procedures and surgical interventions on organs, genitals within 45 days preceding the study.

### 2.3. Sample Collection and Study Settings

The samples were collected using an endocervical brush from the surface of the vaginal part of the cervix (exocervix) and the cervical canal (endocervix—the “transformation zone” of flat and columnar epithelium). These endocervical specimens were preserved in transport medium and sent to the laboratory on the same day.

The cervical smear samples were used to extract HPV DNA for further HPV genotyping, which was performed by utilizing RealBest kit (Vector Best, Koltsovo, Russian Federation) that identifies 12 high-risk HPV types (16, 18, 31, 33, 35, 39, 45, 51, 52, 56, 58, 59) according to the manufacturer’s instructions. The kit was validated in our previous studies [22,23]. The real-time PCR (RT-PCR) was performed with the CFX 96 RealTime PCR machine (Bio-Rad Laboratories, Hercules, CA, USA). Positive and negative controls were used for each PCR reaction. The positivity or negativity of the samples for HPV type were determined according to the manufacturer’s thresholds. The DNA concentration of the samples used was 3.75 ng/μL, resulting in 37.5 ng per well. The data produced were moved into the manufacturer’s software.

Detection of STIs (*Mycoplasma genitalium*, cytomegalovirus, HSV types 1 and 2, *Trichomonas vaginalis*, *Ureaplasma urealyticum*, *Neisseria gonorrhoeae*, *Candida albicans*, *Chlamydia trachomatis*, *Gardnerella vaginalis*, *Mycoplasma chominis*) was performed by the PCR quantitative method using a RealBest extraction 3 kit using the same machine—CFX 96 RealTime PCR, Bio-Rad Laboratories.

### 2.4. Study Variables

To define the factors that increase the risk of high-risk HPV, the next independent variables were examined: social and demographic features of participants (age, ethnicity, and residence); marital status (married, in a committed relationship; single) and past medical history (number of children, delivery, abortions, etc.); number of sexual partners, and data on gynecological health (menarche, age at the sexual debut, gynecological diseases, gynecological surgeries, history of sexually transmitted infections (STIs), etc.).

### 2.5. Statistical Analysis

The baseline characteristics of study participants were compared between groups stratified by HPV status and STI status. Based on the assessment of data distribution and assumptions for statistical tests, group comparisons were conducted using independent samples t-tests, Mann–Whitney U tests for continuous variables, and chi-square or Fischer’s exact tests for categorical variables. Additional intercomparisons were performed to evaluate the distribution of HPV and STI positivity and multiplicity across HPV and STI groups. To assess the associations between individual STIs and HPV status, univariate and multivariate binary logistic regression models were built. Multivariable models were adjusted for age and barrier contraception use, based on the examination of collinearity and correlations between variables which differed significantly across HPV groups and showed potential for confounding or collider bias.

Statistical analyses, as well as the visualization of results, were conducted using Python version: 3.11.12 (NumPy: 2.0.2, Pandas: 2.2.2, Statsmodels: 0.14.4, SciPy: 1.15.3, Scikit-learn: 1.6.1, Matplotlib: 3.10.0, Seaborn: 0.13.2).

### 2.6. Ethical Consideration

This study was conducted following the Declaration of Helsinki and its subsequent modifications and approved by the Local Bioethics Committee of “UMC” Corporate Fund (Minutes No. 2024/02-013 of 10 May 2024). Before sample collection, all participants provided written informed consent. The research team complies with all principles of scientific ethics and biomedical research ethics, and maintains high standards of intellectual integrity when implementing the program. After signing the informed consent form, the patient was included in the study. No personal information about the patients was made available to the investigators during or after the study.

## 3. Results

### 3.1. Study Subjects Description

A total of 396 women unvaccinated against HPV aged 18 to 45 years participated in this prospective cohort study. The median age of HPV-negative women was 34.96 years (interquartile range (IQR): 31.60–40.00), which was significantly higher than that of HPV-positive women (median 33.86 years, IQR: 27.92–37.48, *p* = 0.001). Marital status differed significantly between groups (*p* = 0.0078), with a higher proportion of single women in the HPV-positive group (30.9%) compared to the HPV-negative group (18.7%). Other socio-demographic variables, including education level, BMI, menarche age, menstrual cycle regularity, and mode of delivery, did not differ significantly by HPV status (Table 1). In terms of clinical data and obstetrics medical history, HPV-positive women reported fewer pregnancies (mean 1.51 vs. 2.38, *p* < 0.001) and deliveries (mean 1.2 vs. 1.74, *p* = 0.0002) compared to HPV-negative women. A significant difference was also found in abortion history (*p* = 0.001). The number of abortions was higher among HPV and STI-negative women than among HPV and STI-positive women (0.36 and 0.33 vs. 0.13 and 0.26, respectively). The use of barrier contraception was more frequent among HPV-positive women (41.0%) than HPV-negative women (29.2%; *p* = 0.0171) (Table 1).

### 3.2. STI Prevalence and Associations

Among STI agents, *Mycoplasma hominis* showed a significant association with HPV infection after adjustment for other STIs, age, and the use of barrier contraception (adjusted OR = 2.13, 95% CI: 1.12–4.06, *p* = 0.021). The presence of any STIs, as well as the multiplicity of STIs, was not associated with HPV status in univariate models. No statistically significant associations were found for other organisms such as *Gardnerella vaginalis*, *Chlamydia trachomatis*, *Candida albicans*, or *Cytomegalovirus* (Table 2).

Logistic regression analysis, in which each STI was included as an individual binary predictor for the presence of HPV infection, revealed that only *Mycoplasma hominis* possesses a statistically significant association with HPV status. Specifically, the likelihood of testing positive for HPV in the presence of *Mycoplasma hominis* infection was more than twofold higher, with the result reaching statistical significance (*p* = 0.021). Although *Chlamydia trachomatis* showed a high estimated risk, the finding lacked stability due to a limited number of observations. No other STIs exhibited a significant association with HPV infection.

Age has a slight protective effect (OR = 0.955) in HPV-positive patients, both in a crude model and while adjusted for STIs and barrier contraception.

**Figure 1:**
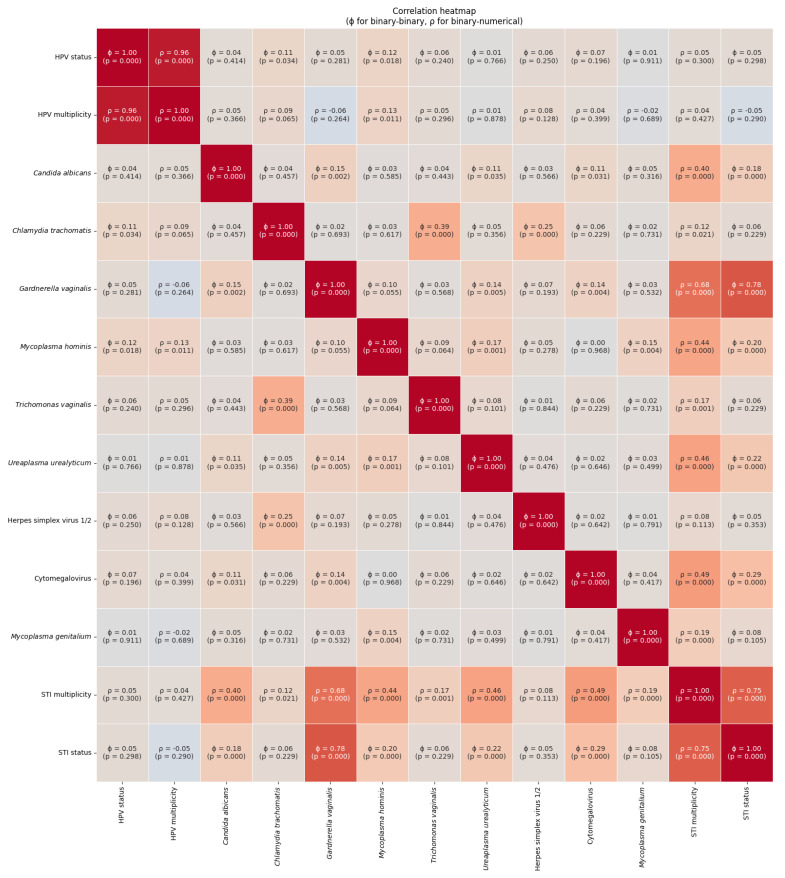
Correlation heatmap of vaginal pathogens.

According to Figure 1, *Mycoplasma hominis* infection possesses a weak, yet statistically significant correlation with both HPV status (ϕ = 0.12, *p* = 0.018) and HPV multiplicity (ρ = 0.13, *p* = 0.011).

Moreover, correlation analysis revealed moderate positive associations between *Chlamydia trachomatis* and *Trichomonas vaginalis* (ϕ = 0.39, *p* < 0.000), as well as weak but significant correlations between *Candida albicans* and *Gardnerella vaginalis* (ϕ = 0.15, *p* = 0.002), and between *Mycoplasma hominis* and *Mycoplasma genitalium* (ϕ = 0.15, *p* = 0.004). These findings indicate the non-random co-occurrence of vaginal pathogens.

## 4. Discussion

The vaginal microflora is a dynamic and self-regulating ecosystem with a significant role in maintaining the female genital tract health [2]. Disruption of the balance between physiologic microbiota allows pathogenic species to grow. In this regard, a special risk is brought by HPV, which has a potential for long-term persistence and thus, causes cervical premalignant lesions and cervical cancer [2,18].

This study found that infection with *Mycoplasma hominis*, the presence of at least one STI, and a greater number of concurrent STIs were all significantly linked to HPV positivity in this Central Asian population. These results are consistent with other studies that have proposed that co-infections may facilitate the persistence of HPV by contributing to mucosal inflammation, altering immune responses, or compromising the integrity of the epithelial barrier [24,25]. Previous studies have noted a link between *Mycoplasma hominis* and HPV infection, suggesting that metabolic by-products produced by *Mycoplasma hominis* may contribute to a pro-inflammatory milieu that supports viral persistence and potentially the development of cervical lesions [26]. The results of our study support this interpretation, especially given the strength of the association observed after adjusting for potential confounding variables. The observed positive association between STI multiplicity and HPV may reflect cumulative exposure to mucosal damage, immune activation, and increased transmission opportunities, especially in sexually active populations with inconsistent condom use [27,28,29]. Moreover, according to research data, condoms do not fully protect from HPV, thus even using barrier methods of contraception women can contract HPV infection, which is in line with our findings [30,31].

The socio-demographic data indicate that HPV positivity is more common among younger and single women, which is in line with patterns reported in international studies [19]. Differences in reproductive history—specifically, the lower number of pregnancies and deliveries among HPV-positive participants—may reflect a combination of behavioral and biological factors that influence both reproductive outcomes and susceptibility to infection. The correlation analysis also revealed the noteworthy co-occurrence of certain STIs, pointing to either overlapping modes of transmission or shared biological environments. In particular, the observed association between *Chlamydia trachomatis* and *Trichomonas vaginalis*—both of which often present without symptoms and are sexually transmitted—underscores the importance of comprehensive STI screening in individuals diagnosed with HPV.

### Strengths and Limitations

One of the key strengths of this study is its prospective cohort design, which enabled structured data collection and the timely assessment of HPV and co-infection status. By comparing HPV-positive and HPV-negative participants, the study offers valuable insights into microbial patterns associated with infection risk. Importantly, the exclusion of vaccinated individuals allowed for an unconfounded analysis of natural HPV infection dynamics. The sample size was relatively large, and the broad range of pathogens tested provided a comprehensive picture of co-infection trends in a population where such data have historically been scarce.

Nonetheless, there are several limitations that should be considered. While significant associations were identified, the study design does not permit conclusions about causality, and longitudinal follow-up would be needed to assess outcomes such as persistence of infection or progression to cervical pathology. Furthermore, the use of PCR-based diagnostics, while sensitive, does not capture microbial load or shifts in community structure, which are increasingly recognized as relevant to disease risk. The study population, drawn from a single screening center, may limit the generalizability of findings to other settings or demographics. In addition, although some behavioral data were collected, variables such as sexual partner dynamics, detailed contraceptive use, and socioeconomic context were not extensively addressed and may represent potential sources of residual confounding.

## 5. Conclusions

This study’s findings indicate a clear link between HPV infection and disruptions in vaginal microflora, particularly with Mycoplasma hominis and the presence of other sexually transmitted infections. Women with HPV were more likely to have one or more STIs, suggesting that microbial imbalance may create a more permissive environment for HPV acquisition or persistence.

These results support the idea that addressing vaginal infections and overall microbial health may be an important, yet often overlooked, part of HPV prevention. Incorporating broader STI screening and treatment into cervical cancer screening programs could improve early detection and reduce long-term risk, especially in populations with limited access to HPV vaccination.

Although this study cannot establish causation, it highlights the need for longer-term research using more advanced microbiome tools to explore how these microbial factors influence the course of HPV infections. Such work could help shape more comprehensive care strategies for women’s reproductive health.

## Figures and Tables

**Table 1:** Socio-demographic and clinical characteristics.

	Statistics	HPV Negative	HPV Positive	STI Negative	STI Positive
Age	Mean ± SD	35.25 ± 6.18	33.17 ± 6.60	35.50 ± 6.19	34.24 ± 6.44
	Median [Q1–Q3]	34.96 [31.60–40.00]	33.86 [27.92–37.48]	35.66 [31.40–39.99]	34.26 [30.20–38.64]
	Min–Max	19.93–45.88	19.90–46.03	19.98–45.87	19.90–46.03
	Shapiro–Wilk (*p*)	0.0003	0.0197	0.0789	0.0001
	Levene’s *p*	0.3625		0.7327	
	Statistic (*p*)	t = 3.13	*p* = 0.0019	t = 1.63	*p* = 0.1038
Ethnicity	Kazakh	236 (91.8%)	125 (89.9%)	82 (93.2%)	279 (90.6%)
	Russian	12 (4.7%)	8 (5.8%)	2 (2.3%)	18 (5.8%)
	Uzbek	0 (0.0%)	1 (0.7%)	0 (0.0%)	1 (0.3%)
	Ukranian	2 (0.8%)	0 (0.0%)	1 (1.1%)	1 (0.3%)
	Uighur	1 (0.4%)	0 (0.0%)	0 (0.0%)	1 (0.3%)
	Korean	1 (0.4%)	0 (0.0%)	1 (1.1%)	0 (0.0%)
	Armenian	2 (0.8%)	0 (0.0%)	0 (0.0%)	2 (0.6%)
	Bashkir	1 (0.4%)	0 (0.0%)	0 (0.0%)	1 (0.3%)
	Tatar	1 (0.4%)	2 (1.4%)	0 (0.0%)	3 (1.0%)
	German	1 (0.4%)	1 (0.7%)	0 (0.0%)	2 (0.6%)
	Georgian	0 (0.0%)	1 (0.7%)	1 (1.1%)	0 (0.0%)
	Macedonian	0 (0.0%)	1 (0.7%)	1 (1.1%)	0 (0.0%)
	Statistic (*p*)	Chi-square: 11.09	*p* = 0.4354	Chi-square: 16.03	*p* = 0.1400
Education	Incomplete secondary	2 (0.8%)	1 (0.7%)	1 (1.1%)	2 (0.6%)
	Secondary professional	42 (16.3%)	17 (12.2%)	13 (14.8%)	46 (14.9%)
	Bachelor	178 (69.3%)	98 (70.5%)	61 (69.3%)	215 (69.8%)
	Masters	32 (12.5%)	21 (15.1%)	11 (12.5%)	42 (13.6%)
	PhD	3 (1.2%)	2 (1.4%)	2 (2.3%)	3 (1.0%)
	Statistic (*p*)	Chi-square: 1.58	*p* = 0.8130	Chi-square: 1.2	*p* = 0.8784
Marital status	Single	48 (18.7%)	43 (30.9%)	20 (22.7%)	71 (23.1%)
	Married	184 (71.6%)	77 (55.4%)	60 (68.2%)	201 (65.3%)
	Divorced	22 (8.6%)	18 (12.9%)	8 (9.1%)	32 (10.4%)
	Widow	3 (1.2%)	1 (0.7%)	0 (0.0%)	4 (1.3%)
	Statistic (*p*)	Chi-square: 11.39	*p* = 0.0098	Chi-square: 1.35	*p* = 0.7175
BMI	Mean ± SD	23.85 ± 4.62	23.36 ± 4.56	23.93 ± 4.29	23.61 ± 4.69
	Median [Q1–Q3]	23.14 [20.57–26.35]	22.31 [20.26–25.56]	23.20 [20.89–26.45]	22.86 [20.39–25.84]
	Min–Max	15.78–43.26	14.88–42.80	16.65–43.26	14.88–42.80
	Shapiro–Wilk (*p*)	0.0000	0.0000	0.0000	0.0000
	Levene’s *p*	0.8936		0.3703	
	Statistic (*p*)	t = 1.03	*p* = 0.3037	t = 0.58	*p* = 0.5639
Menarche	Mean ± SD	13.41 ± 1.37	13.25 ± 1.22	13.15 ± 1.37	13.41 ± 1.30
	Median [Q1–Q3]	13.00 [13.00–14.00]	13.00 [13.00–14.00]	13.00 [12.00–14.00]	13.00 [13.00–14.00]
	Min–Max	10.00–18.00	10.00–17.00	10.00–17.00	10.00–18.00
	Shapiro–Wilk (*p*)	0.0000	0.0000	0.0004	0.0000
	Levene’s *p*	0.0454		0.5541	
	Statistic (*p*)	U = 18,825.00	*p* = 0.3537	t = −1.66	*p* = 0.0973
Duration of menstrual cycle	Mean ± SD	30.37 ± 9.88	29.01 ± 3.73	30.01 ± 8.41	29.86 ± 8.26
	Median [Q1–Q3]	28.00 [28.00–30.00]	28.00 [28.00–30.00]	28.00 [28.00–30.00]	28.00 [28.00–30.00]
	Min–Max	21.00–100.00	20.00–60.00	23.00–90.00	20.00–100.00
	Shapiro–Wilk (*p*)	0.0000	0.0000	0.0000	0.0000
	Levene’s *p*	0.0716		0.9024	
	Statistic (*p*)	t = 1.57	*p* = 0.1182	t = 0.15	*p* = 0.8778
Regularity of menstrual cycle	Regular	239 (93.0%)	132 (95.0%)	82 (93.2%)	289 (93.8%)
	Irregular	18 (7.0%)	7 (5.0%)	6 (6.8%)	19 (6.2%)
	Statistic (*p*)	Chi-square: 0.3	*p* = 0.5809	Chi-square: 0.0	*p* = 1.0000
Pain during menstruation	Painless	255 (99.2%)	138 (99.3%)	87 (98.9%)	306 (99.4%)
	Painful	2 (0.8%)	1 (0.7%)	1 (1.1%)	2 (0.6%)
	Statistic (*p*)	Fisher: 0.92	*p* = 1.0000	Fisher: 0.57	*p* = 0.5305
Volume of menses	Light	7 (2.7%)	3 (2.2%)	2 (2.3%)	8 (2.6%)
	Medium	250 (97.3%)	136 (97.8%)	86 (97.7%)	300 (97.4%)
	Statistic (*p*)	Fisher: 1.27	*p* = 1.0000	Fisher: 0.87	*p* = 1.0000
Number of sexual partners	Mean ± SD	1.57 ± 1.79	2.18 ± 5.42	1.61 ± 1.57	1.83 ± 3.91
	Median [Q1–Q3]	1.00 [1.00–1.00]	1.00 [1.00–2.00]	1.00 [1.00–1.00]	1.00 [1.00–1.00]
	Min–Max	0.00–20.00	0.00–60.00	0.00–9.00	0.00–60.00
	Shapiro–Wilk (*p*)	0.0000	0.0000	0.0000	0.0000
	Levene’s *p*	0.0919		0.6804	
	Statistic (*p*)	t = −1.65	*p* = 0.0991	t = −0.51	*p* = 0.6100
Number of pregnancies	Mean ± SD	2.38 ± 1.91	1.51 ± 1.60	2.19 ± 1.89	2.04 ± 1.84
	Median [Q1–Q3]	2.00 [1.00–4.00]	1.00 [0.00–2.00]	2.00 [1.00–3.00]	2.00 [0.00–3.00]
	Min–Max	0.00–8.00	0.00–9.00	0.00–8.00	0.00–9.00
	Shapiro–Wilk (*p*)	0.0000	0.0000	0.0000	0.0000
	Levene’s *p*	0.0128		0.6853	
	Statistic (*p*)	U = 22,720.00	*p* = 0.0000	t = 0.69	*p* = 0.4909
Number of deliveries	Mean ± SD	1.74 ± 1.39	1.20 ± 1.26	1.62 ± 1.34	1.53 ± 1.38
	Median [Q1–Q3]	2.00 [0.00–3.00]	1.00 [0.00–2.00]	2.00 [0.75–2.00]	1.00 [0.00–2.00]
	Min–Max	0.00–5.00	0.00–5.00	0.00–5.00	0.00–5.00
	Shapiro–Wilk (*p*)	0.0000	0.0000	0.0000	0.0000
	Levene’s *p*	0.0737		0.4127	
	Statistic (*p*)	t = 3.81	*p* = 0.0002	t = 0.56	*p* = 0.5776
Number of abortions	Mean ± SD	0.36 ± 0.74	0.13 ± 0.43	0.33 ± 0.74	0.26 ± 0.63
	Median [Q1–Q3]	0.00 [0.00–0.00]	0.00 [0.00–0.00]	0.00 [0.00–0.00]	0.00 [0.00–0.00]
	Min–Max	0.00–4.00	0.00–3.00	0.00–4.00	0.00–3.00
	Shapiro–Wilk (*p*)	0.0000	0.0000	0.0000	0.0000
	Levene’s *p*	0.0009		0.4012	
	Statistic (*p*)	U = 20,258.00	*p* = 0.0011	t = 0.84	*p* = 0.4012
Number of miscarriages	Mean ± SD	0.25 ± 0.59	0.16 ± 0.49	0.18 ± 0.54	0.22 ± 0.56
	Median [Q1–Q3]	0.00 [0.00–0.00]	0.00 [0.00–0.00]	0.00 [0.00–0.00]	0.00 [0.00–0.00]
	Min–Max	0.00–3.00	0.00–3.00	0.00–3.00	0.00–3.00
	Shapiro–Wilk (*p*)	0.0000	0.0000	0.0000	0.0000
	Levene’s *p*	0.1391		0.5317	
	Statistic (*p*)	t = 1.48	*p* = 0.1391	t = −0.63	*p* = 0.5317
Mode of delivery (History of C-section)	Only spontaneous vaginal	230 (89.5%)	126 (90.6%)	77 (87.5%)	279 (90.6%)
	At least one C-section	27 (10.5%)	13 (9.4%)	11 (12.5%)	29 (9.4%)
	Statistic (*p*)	Chi-square: 0.04	*p* = 0.8502	Chi-square: 0.42	*p* = 0.5181
Abortions	no	198 (77.0%)	125 (89.9%)	70 (79.5%)	253 (82.1%)
	yes	59 (23.0%)	14 (10.1%)	18 (20.5%)	55 (17.9%)
	Statistic (*p*)	Chi-square: 9.12	*p* = 0.0025	Chi-square: 0.16	*p* = 0.6904
Ectopic pregnancy	no	248 (96.5%)	137 (98.6%)	84 (95.5%)	301 (97.7%)
	yes	9 (3.5%)	2 (1.4%)	4 (4.5%)	7 (2.3%)
	Statistic (*p*)	Fisher: 0.4	*p* = 0.3416	Fisher: 0.49	*p* = 0.2716
Barrier contraception	no	182 (70.8%)	82 (59.0%)	58 (65.9%)	206 (66.9%)
	yes	75 (29.2%)	57 (41.0%)	30 (34.1%)	102 (33.1%)
	Statistic (*p*)	Chi-square: 5.16	*p* = 0.0232	Chi-square: 0.0	*p* = 0.9659
Hormonal contraception	no	249 (96.9%)	136 (97.8%)	84 (95.5%)	301 (97.7%)
	yes	8 (3.1%)	3 (2.2%)	4 (4.5%)	7 (2.3%)
	Statistic (*p*)	Fisher: 0.69	*p* = 0.7536	Fisher: 0.49	*p* = 0.2716
Any contraception	no	174 (67.7%)	79 (56.8%)	54 (61.4%)	199 (64.6%)
	yes	83 (32.3%)	60 (43.2%)	34 (38.6%)	109 (35.4%)
	Statistic (*p*)	Chi-square: 4.16	*p* = 0.0414	Chi-square: 0.19	*p* = 0.6647
Any gynecological diseases	no	177 (68.9%)	93 (66.9%)	56 (63.6%)	214 (69.5%)
	yes	80 (31.1%)	46 (33.1%)	32 (36.4%)	94 (30.5%)
	Statistic (*p*)	Chi-square: 0.08	*p* = 0.7736	Chi-square: 0.82	*p* = 0.3637
Cervical erosion	no	136 (52.9%)	82 (59.0%)	53 (60.2%)	165 (53.6%)
	yes	121 (47.1%)	57 (41.0%)	35 (39.8%)	143 (46.4%)
	Statistic (*p*)	Chi-square: 1.11	*p* = 0.2919	Chi-square: 0.97	*p* = 0.3244
STI status	STI negative	53 (20.6%)	35 (25.2%)	–	–
	STI positive	204 (79.4%)	104 (74.8%)		
	Statistic (*p*)	Chi-square: 0.84	*p* = 0.3604		
STI multiplicity	Mean ± SD	1.27 ± 0.94	1.44 ± 1.19	–	–
	Median [Q1–Q3]	1.00 [1.00–2.00]	1.00 [0.50–2.00]		
	Min–Max	0.00–4.00	0.00–5.00		
	Shapiro–Wilk (*p*)	0.0000	0.0000		
	Levene’s *p*	0.0011			
	Statistic (*p*)	U = 16,784.50	*p* = 0.2995		
HPV status	HPV negative	–	–	53 (60.2%)	204 (66.2%)
	HPV positive			35 (39.8%)	104 (33.8%)
	Statistic (*p*)			Chi-square: 0.84	*p* = 0.3604
HPV multiplicity	Mean ± SD	–	–	0.60 ± 0.88	0.54 ± 0.93
	Median [Q1–Q3]			0.00 [0.00–1.00]	0.00 [0.00–1.00]
	Min–Max			0.00–4.00	0.00–6.00
	Shapiro–Wilk (*p*)			0.0000	0.0000
	Levene’s *p*			0.5684	
	Statistic (*p*)			t = 0.57	*p* = 0.5684

**Table 2:** Association between vaginal pathogens with HPV infection.

STI Column	Cases in HPV− (*n* = 257)	Cases in HPV+ (*n* = 139)	Crude OR	Crude OR 2.5%	Crude OR 97.5%	Crude OR *p*-Value	Adjusted OR	Adjusted OR 2.5%	Adjusted OR 97.5%	Adjusted OR *p*-Value
*Candida albicans*	23 (8.94%)	16 (11.51%)	1.323	0.674	2.598	0.415	1.179	0.577	2.409	0.651
*Chlamydia trachomatis*	1 (0.39%)	4 (2.88%)	7.585	0.839	68.539	0.071	8.495	0.723	99.795	0.089
*Gardnerella vaginalis*	180 (70.04%)	90 (64.75%)	0.786	0.507	1.218	0.281	0.647	0.402	1.039	0.072
*Mycoplasma hominis*	25 (9.73%)	25 (17.99%)	2.035	1.119	3.701	0.020	2.132	1.120	4.057	0.021
*Trichomonas vaginalis*	2 (0.78%)	3 (2.16%)	2.813	0.464	17.036	0.261	1.944	0.226	16.697	0.545
*Ureaplasma urealyticum*	36 (14.01%)	21 (15.11%)	1.093	0.610	1.957	0.766	1.023	0.549	1.905	0.943
Herpes simplex virus 1/2	1 (0.39%)	2 (1.44%)	3.737	0.336	41.585	0.284	1.271	0.087	18.536	0.861
Cytomegalovirus	52 (20.23%)	36 (25.90%)	1.378	0.847	2.241	0.196	1.509	0.902	2.523	0.117
*Mycoplasma genitalium*	6 (2.33%)	3 (2.16%)	0.923	0.227	3.748	0.911	0.622	0.140	2.757	0.532
STI status	204 (79.4%)	104 (74.8%)	0.772	0.474	1.257	0.299	–	–	–	–
STI multiplicity	1.27 ± 0.94	1.44 ± 1.19	1.170	0.960	1.427	0.120	–	–	–	–
Barrier contraception	75 (29.18%)	57 (41.01%)	1.687	1.095	2.598	0.018	1.528	0.958	2.436	0.075
Age	35.25 ± 6.18	33.17 ± 6.60	0.950	0.919	0.982	0.002	0.955	0.921	0.990	0.013

## Data Availability

The data can be shared up on request.
